# Draft genome sequences of two strains of *Staphylococcus aureus* isolated from mastitis-infected camel in Kajiado County, Kenya

**DOI:** 10.1128/MRA.00254-23

**Published:** 2023-07-27

**Authors:** Kamau Mwangi, Rachael Gachogo, Ednah Masila, Irene Ogali, Nathan Langat, Ruth Onywera, Vincent Malonza, Hezron Wesonga, Monicah Maichomo, Edwin Murungi

**Affiliations:** 1 Veterinary Research Institute, Kenya Agricultural and Livestock Research Organization, Nairobi, Kenya; 2 Division of Immunology, Department of Human Pathology, University of Cape Town, Cape Town, Western Cape, South Africa; 3 Department of Medical Biochemistry, Kisii University, Kisii, Kenya; Indiana University, Bloomington, Bloomington, Indiana, USA

**Keywords:** mastitis, camel, veterinary vaccine development, *Staphylococcus aureus*, milk

## Abstract

We report the draft genome sequences of two *Staphylococcus aureus* strains isolated from a mastitis-infected camel in Kajiado County, Kenya. The 2,739,512-bp and 3,025,943-bp draft genomes coding for 2,577 and 2,889 protein sequences, respectively, provide invaluable data for the computational design of a camel mastitis subunit vaccine.

## ANNOUNCEMENT

Camels are a vital source of livelihood and nutrition for populations in the arid and semi-arid regions of Africa (1, 2) that are increasingly being ravaged by severe droughts because of climate change. Camel mastitis due to several bacteria species, including *Staphylococcus aureus,* causes colossal economic losses for pastoralists (3).

We report the draft genome sequences of two strains of *S. aureus* (KALRO-12HR and KALRO-4HL) isolated from a mastitis-infected nomadic camel in Kajiado County, Kenya. Prior to milk samples collection, the teats were thoroughly washed with clean water and swabbed with 70% ethanol. Milk samples from individual teats were collected in sterile falcon tubes and transported for approximately 60 km in a vaccination cooler box to Kenya Agricultural and Livestock Research Organization (KALRO) Muguga Center for storage at −20°C. The samples were subsequently cultured by streaking on blood agar containing 5% defribinated sheep blood followed by overnight incubation at 37°C (4). Growth scoring, catalase testing, and Gram staining were performed to delineate *Staphylococcus* spp. with catalase-positive colonies appearing as Gram-positive cocci clusters capable of growing in tryptic soy broth (TSB) containing 10% glycerol presumed to be *Staphylococcus* spp. Genomic DNA was extracted from overnight TSB culture using QIAamp DNA Kit (Qiagen, Netherlands), and libraries were prepared using Nextera DNA Flex Library Preparation Kit (Illumina, San Diego, CA, USA) according to manufacturers’ protocols. The library fragment was determined using Qubit dsDNA HS Assay Kit (Thermo Scientific, Waltham, MA, USA), and the library sequenced on an Illumina MiSEQ platform with a paired-end 300-bp MiSeq reagent kit v3 (Illumina, USA) to give 5,540,333 and 5,396,826 paired-end reads, respectively, at a sequencing depth of 142× and 138×, respectively. The quality of the sequencing reads was ascertained using FASTQC v0.11.5 (https://www.bioinformatics.babraham.ac.uk/projects/fastqc/), and trimming undertaken using TRIMMOMATIC v0.39 (5). Genome assembly was performed using SPADES v3.14 and quality evaluated using QUAST v5.2.0 (6). Annotation of assembled genomes was performed using PGAP v6.4 (7). Default parameters were used for all software unless otherwise specified. The 2,739,512-bp KALRO-12HR strain with an N_50_ of 118,743 bp and GC content of 32.5% contains 2,577 protein coding genes, 30 tRNAs, and 5 rRNAs. On the other hand, the 3,025,943-bp KALRO-4HL strain with an N_50_ of 89,800 and GC content of 33% comprises 2,889 protein coding genes, 34 tRNAs, and 9 rRNAs.

Using VaxiJen (8), IgPred (9), and AllerTOP v.2 (10), we have identified several potentially viable antigenic vaccine candidates. Sub-cellular localization and transmembrane helices were predicted using DeepLoc (11) and DeepTMHMM (12), respectively (Table 1). Our results provide an invaluable data resource for further investigations aimed at the development of a novel subunit vaccine for camel mastitis.

**TABLE 1:** Putative vaccine candidates for KALRO-12HR and KALRO-4HL *S. aureus* strains

Strain	NCBI sequence accession	VaxiJen score	Subcellular localization	Transmembrane helices	IgPred prediction	AllerTOP prediction
4HL	MDF3296843.1	1.5523	Cellwall	0	IgG epitope	Probable non-allergen
	MDF3297031.1	1.5055	Extracellular	0	IgG epitope	Probable non-allergen
	MDF3296249.1	1.2907	Extracellular	1	IgG epitope	Probable non-allergen
	MDF3296104.1	1.2320	Extracellular	0	IgG epitope	Probable non-allergen
	MDF3295251.1	1.2194	Unknown	0	IgG epitope	Probable non-allergen
	MDF3296239.1	1.0896	Cellwall	1	IgG epitope	Probable non-allergen
	MDF3295281.1	1.0537	Cellwall	1	IgG epitope	Probable non-allergen
12HR	MDF3345085.1	1.4003	Extracellular	0	IgG epitope	Probable non-allergen
	MDF3344957.1	1.0859	Extracellular	1	IgG epitope	Probable non-allergen
	MDF3344617.1	0.8172	Unknown	1	IgG epitope	Probable non-allergen
	MDF3343733.1	0.8171	Cellwall	1	IgG epitope	Probable non-allergen

## Data Availability

This Whole Genome Shotgun project has been deposited at DDBJ/ENA/GenBank under accessions JARJBG000000000 and JARJBH000000000. Raw sequence data are available at SRA under accession PRJNA943585).
